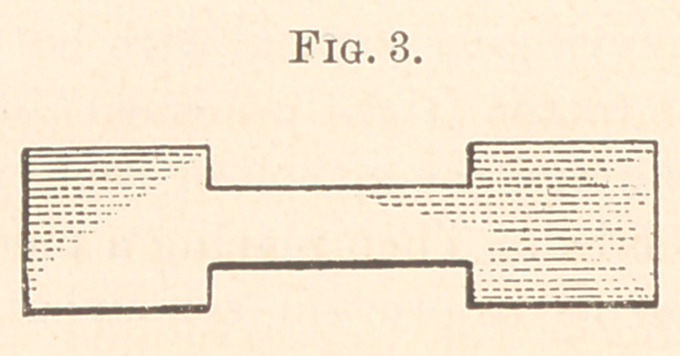# Hemorrhages after Extraction of Teeth in Hemorrhagic Diathesis and Spontaneous Anæmia

**Published:** 1894-10

**Authors:** D. A. Rosenthal

**Affiliations:** Philadelphia


### HEMORRHAGES AFTER EXTRACTION OF TEETH IN HEMORRHAGIC DIATHESIS AND SPONTANEOUS ANÆMIA.

BY D. A. ROSENTHAL, D.D.S., PHILADELPHIA.

The extraction of teeth, being an operation that nearly every dentist is called upon to perform, oftentimes results in hemorrhages requiring most energetic treatment to check.

Especially is this true in cases of "hemorrhagic diathesis and spontaneous anæmia," where the blood will continue oozing in spite of all local treatments.

It will be found necessary and of great value to administer remedies tending to correct the abnormal condition of the blood, and which would promote contractility of the blood-vessels.

Depressing the action of the heart is, under all circumstances, an admirable step towards the checking of the hemorrhage.

Veratrum viride, gtt. iv in a tablespoonful of water, every three hours, will be found beneficial. Twelve drops will generally cause the desired effect.

In "spontaneous anæmia," the proportion of red globules may sink as low as six or four per cent., which makes it about seven to nine per cent. less than the normal proportion of red globules to the whole blood.

The red globules, when lost, are regenerated slowly, and when hemorrhages are frequent, the effect on the quality may become serious. The blood becomes watery, rendering it very difficult to control hemorrhage.

In cases of spontaneous anæmia, the vital powers of patients are very much reduced. Patients exhibit marked debility, and it requires careful treatment to effect a cure.

The following tonic, as recommended by Dr. A. H. Smith, is very good.

℞ Hydrargyri chloridi corrosivum, gr. j;
Liq. arsenici chloridi, gtt. xlviij;
Tinct. ferri chloridi,
Acidi hydrochlorici dil., āā f℥iv;
Syrupi, f℥iij;
Aqua, q.s. ad. f℥vj. M.

Sig.—A dessertspoonful in a wineglassful of water, after each meal.

Doing considerable extraction, I had a few cases of profuse hemorrhage, and the following treatment resulted in its immediate control:

Illustrative example:

Miss B., about seventeen years old, came to my office, and had the second inferior molar extracted.

The tooth was very much affected by caries, and its removal was decided upon. The tooth had an inward curve at the root, which made it rather difficult to extract without a little more than the usual force, and possibility of fracture.

After the tooth was removed, the patient complained of slight pain, undoubtedly due to the extra movements occasioned by the caries at the roots. I also noticed that the hemorrhage was rather unusual, but thinking that it may have been due to the inflammation of dental membrane, I simply squeezed together the pressed-apart alveolar process, and dismissed patient with usual directions to rinse mouth with salt water.

Two days after, patient came complaining of profuse hemorrhage after leaving my office, asserting that she nearly died from hemorrhage after the extraction of a tooth while in Italy. Her father also had a similar experience.

The patient was weak, felt faint, had cold hands, was suffering from muscular and general debility, and looked very anæmic.

I had the following prescription filled, and after having administered one dose, I proceeded to the local and mechanical treatment, as will be seen below.

℞ Tinct. digitalis, f ʒ iss ;

Tinct. catechu, f℥j ;

Extr. ergotæ fl., q.s. ad. f℥ij. M.

Sig.—A dessertspoonful every two hours.

After syringing sockets with hot water I dipped a few pieces of absorbent cotton (tightly formed) in tannic acid, and placed them one by one into the alveoli or root cavities, until they were tightly and fully filled. Then placing a piece of rubber dam over the filled surface so as to prevent somewhat the tannic acid from mixing with the saliva, I placed a piece of warmed wax with "plug-retainer," as I call it, asking patient to bite, always observing that such should be normal. After this I made a firm bandage as modified by Professor J. E. Garretson, which is as follows:

I took a strip one and a half inches wide, and one and a half

FIG. 1.

yards long of rubber material; placed centre of strip against chin of patient; carried the ends up, crossed on forehead; carried around sides of cranium, crossed at the occiput; carried forward, and tied in front of the chin as represented in cut (Fig. 1).

Saw patient in two hours, and after removing bandage, found slight sign of hemorrhage. At this time patient took some liquid food and another dose of medicine; rebandaged and dismissed her. When seen next morning I removed all dressing, and found that hemorrhage has entirely ceased.

With this method of treatment I have been successful in stopping every case of hemorrhage I had to deal with. It is well to observe that the pieces of cotton be placed in the root-sockets in such a manner that the bony part partitioning them off should not serve as a preventive of packing them solid, as failure in reaching the depth of root-cavities will serve as an obstacle in obtaining satisfactory results.

FIG. 2.

FIG. 3.

This plug-retainer (Fig. 2) will prove of admirable value in helping to retain all applications in cases of hemorrhage after the extraction of teeth. It can easily be adapted to any part of the mouth.

You can make it yourself at any desired moment, but it is better to have one on hand so as not to lose time when it is wanted.

Take a piece of metal about twenty-four gauge, and cut into shape of Fig. 3, bending the wide ends, slightly inclining them forward, as represented in Fig. 2.

Always place a small pad of cotton over cross piece, and patient is ready to bite. It will sooner or later do the dentist great service if he will find room in his dental cabinet for a roll of unbleached muslin about one and a half inches wide, impression-wax, plug-retainer, and pellets of cotton dipped in tannic acid.

## Figures and Tables

**Fig. 1. f1:**
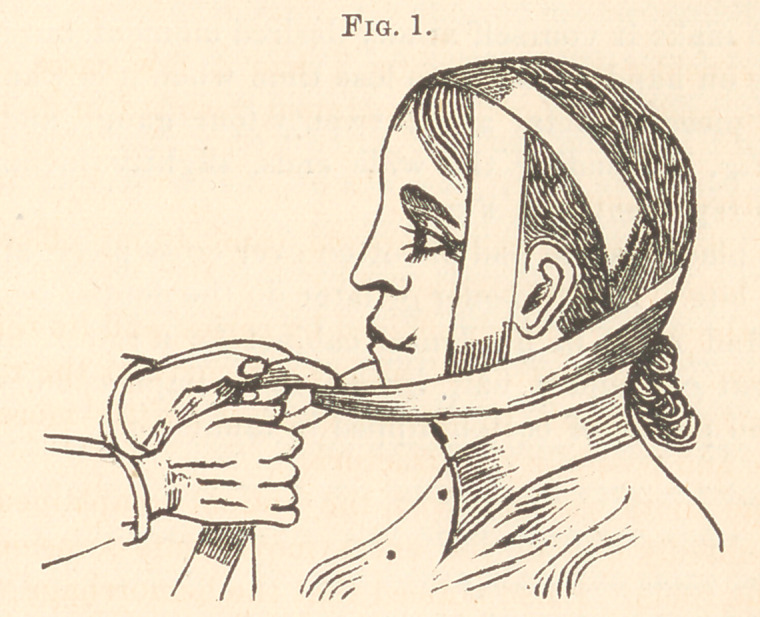


**Fig. 2. f2:**
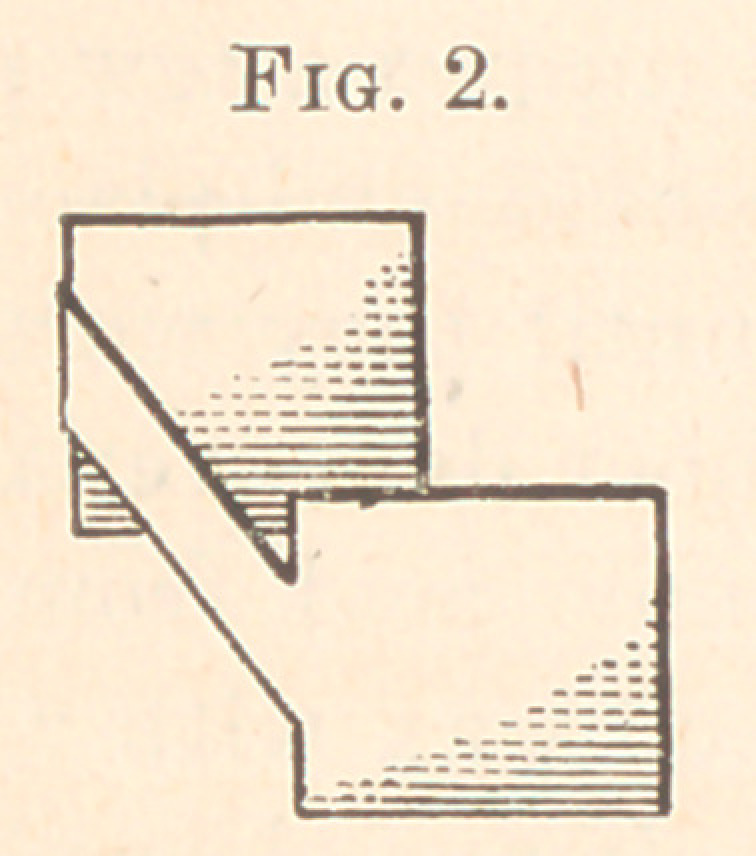


**Fig. 3. f3:**